# Web-Based Virtual Patients in Nursing Education: Development and Validation of Theory-Anchored Design and Activity Models

**DOI:** 10.2196/jmir.2556

**Published:** 2014-04-10

**Authors:** Carina Georg, Nabil Zary

**Affiliations:** ^1^Center for Learning and Knowledge (CLK)Department of Learning, Informatics, Management and Ethics ( LIME)Karolinska InstitutetStockholmSweden; ^2^Department of Neurobiology, Care Science and SocietyDivision of NursingKarolinska InstitutetHuddingeSweden

**Keywords:** virtual patient, patient simulation, nursing education, clinical reasoning, e-learning, simulation technology

## Abstract

**Background:**

Research has shown that nursing students find it difficult to translate and apply their theoretical knowledge in a clinical context. Virtual patients (VPs) have been proposed as a learning activity that can support nursing students in their learning of scientific knowledge and help them integrate theory and practice. Although VPs are increasingly used in health care education, they still lack a systematic consistency that would allow their reuse outside of their original context. There is therefore a need to develop a model for the development and implementation of VPs in nursing education.

**Objective:**

The aim of this study was to develop and evaluate a virtual patient model optimized to the learning and assessment needs in nursing education.

**Methods:**

The process of modeling started by reviewing theoretical frameworks reported in the literature and used by practitioners when designing learning and assessment activities. The Outcome-Present State Test (OPT) model was chosen as the theoretical framework. The model was then, in an iterative manner, developed and optimized to the affordances of virtual patients. Content validation was performed with faculty both in terms of the relevance of the chosen theories but also its applicability in nursing education. The virtual patient nursing model was then instantiated in two VPs. The students’ perceived usefulness of the VPs was investigated using a questionnaire. The result was analyzed using descriptive statistics.

**Results:**

A virtual patient Nursing Design Model (vpNDM) composed of three layers was developed. Layer 1 contains the patient story and ways of interacting with the data, Layer 2 includes aspects of the iterative process of clinical reasoning, and finally Layer 3 includes measurable outcomes. A virtual patient Nursing Activity Model (vpNAM) was also developed as a guide when creating VP-centric learning activities. The students perceived the global linear VPs as a relevant learning activity for the integration of theory and practice.

**Conclusions:**

Virtual patients that are adapted to the nursing paradigm can support nursing students’ development of clinical reasoning skills. The proposed virtual patient nursing design and activity models will allow the systematic development of different types of virtual patients from a common model and thereby create opportunities for sharing pedagogical designs across technical solutions.

## Introduction

### Background

One of the main goals in health care education is to supply society with knowledgeable, up-to-date, and skilled professionals [1]. The societal and health care expectations in providing adequate learning experiences can be overwhelming for both learners and educators [2], and the health care environment is dynamic and continuously changing with fewer patients in hospitals staying shorter periods and with specialized health care needs. There are also technological advances, financial challenges, and numerous regulations [3]. The teaching and learning of professional skills must be conducted without waiving patient safety. Therefore, this continuously changing health care environment requires new models for training health care professionals [4]. One challenge is how to teach students to apply their knowledge when they are dealing with clinical problems [5]. Technology-enhanced simulation is one possible solution [4]. Different types of simulation modalities as a teaching method in health care education have developed rapidly during the last decade [6,7]. Simulation offers a safe and realistic environment in which to learn, practice, and make mistakes without direct contact with and risk of harm to patients [8]. A type of computer-based simulation called virtual patients (VPs) has been proposed to support nursing students in their acquisition of scientific knowledge as a way to integrate theory and practice and promote clinical reasoning.

### Challenges in Nursing Education

Nursing as a profession is at a turning point. Nurses’ work has evolved as a result of a profound change in health care environments, science and technology, and the settings and nature of nursing practice. These changes have important implications for nursing education [3].

Nursing education varies in different parts of the world [9]. The variation lies in the organization, content, and quality of education [10]. In Sweden, the nursing study program covers a total of 180 credits and is conducted at a university level leading to a Bachelor of Science degree in Nursing. After graduation, nursing students become registered nurses (RNs) and obtain a license to work. The goal for nursing education is to support students in acquiring the knowledge and competencies required to provide a good quality of care. The newly graduated nurse should be prepared to practice safely, accurately, and compassionately in various settings. To be able to conduct safe and effective care, the newly graduated nurse must be proficient both in practical knowledge and science and also in a wide range of skills such as clinical reasoning [3]. The development of clinical performance and competencies among nursing students continues to challenge nurse educators, specifically when trying to link nursing theory and evidence-based clinical practice for the student [2,3].

A first step for students toward achieving competencies is to acquire theoretical scientific knowledge and then use this theoretical knowledge in patient-related practice. However, research has shown that nursing students find it difficult to translate and apply their theoretical knowledge in a clinical context [3]. Therefore, a challenge in nursing education is to find ways of preparing nursing students to manage, interpret data, evaluate nursing activities and interventions, and to translate theoretical knowledge to the clinical context. Nurses must think and reason across diverse contexts, which requires the development of strong clinical reasoning skills [11]. Nursing care also integrates knowledge from other disciplines such as medicine and behavioral science [3]. The students usually develop and integrate various aspects of knowledge during their encounters with patients while they observe, understand, and argue for their choices. Theory and reflection form the basis for nursing knowledge that is the prerequisite for good management of the patient [11]. Additionally, critical thinking is an essential skill that requires both cognitive and metacognitive capabilities [12].

Many courses in nursing have learning outcomes that include clinical decision making, clinical reasoning, and critical thinking [13]. However, the literature reports limited skills in clinical reasoning among nursing students. Therefore, nursing students must learn how to apply theoretical knowledge acquired in classrooms to the clinical context. Nursing educators have to develop teaching and learning approaches that enable students to acquire knowledge and skills and to then apply theoretical knowledge in the clinical context.

### Clinical Reasoning in Nursing

Clinical reasoning (CR) is not unique to nursing—all professionals use CR to reach decisions [14]. CR is a complex task [15], and in this study, we have adopted Higgs’ definition:

Clinical reasoning (or practice decision making) is a context-dependent way of thinking and decision making in professional practice to guide practice actions. It involves the construction of narratives to make sense of the multiple factors and interests pertaining to the current reasoning task. It occurs within a set of problem spaces informed by the practitioner´s unique frames of reference, workplace context and practice model, as well as by the patient’s or clients’ contexts. It utilizes core dimensions of practice knowledge, reasoning and metacognition and draws on these capacities in others. Decision making within clinical reasoning occurs at micro, macro and meta-levels and may be individually or collaboratively conducted. It involves metaskills of critical conversations, knowledge generation, practice model authenticity and reflexivity.

16

CR is a logical process where nurses and other health professionals collect cues, process information, and come to an understanding about patients’ situations or problems, plan and implement interventions, and evaluate outcomes [17]. CR represents the essence of nursing practice [12]. There are several different types of clinical reasoning, including problem-effect, which means identifying the problem, seeing the factors influencing the problem, and finding solutions to the problem [15]. This approach helps nurses identify nursing problems and prescribe and implement nursing actions [18].

In terms of CR, a nurse’s focus is the process of care and the well-being of the patient. A nurse evaluates both subjective factors (like descriptions of symptoms) and objective factors (like lab data). The nurse should try to see possible patterns and decide on, plan, and perform patient care, as well as continuously assess care interventions and status of the patient. A competent nurse that provides safe patient care possesses skills in caring sciences and knowledge in both medical and behavioral sciences [19].

CR is widely acknowledged as a fundamental part of health professionals’ education, but there is still a need for the development of innovative teaching and learning methods that enhance CR skills among nursing students [3,18,20]. Virtual patients (VPs) have been proposed as a learning activity that can support students to integrate theory and practice in their development of clinical reasoning skills [21,22].

### Virtual Patients

VPs can take many different forms [23] and can be realized using a wide range of presentations, styles, and configurations [24]. There are also several definitions for the concept of the virtual patient [21], and the term VP is often used in an ambiguous manner [25]. In this study, we adopted Ellaway’s definition: “An interactive computer simulation of real-life clinical scenarios for the purpose of health care and medical training, education or assessment” [26].

VPs can be designed in different ways and are often dependent on the technical affordances of the system used to author them [25]. Most VPs have common features including medical history taking, physical examinations, lab/imaging tests, as well as features for suggesting an appropriate diagnosis and treatment [21]. An essential characteristic of VPs is the interactive interface that enables the user to query the patient and receive a patient response supplied by the computer [21,22,27].

A particular strength of VPs is that they seem to support learning on clinical reasoning and decision making. Students may be exposed to a large number of VP cases in a safe and controlled environment [21,24]. Clinical learning experience is difficult to standardize and schedule in a reproducible manner. However, VPs can provide exactly the same experience repeatedly and also allow students to revisit their actions during the interaction with the virtual patient, and then compare them with the best practice protocol. VPs also facilitate a venue for safe and repetitive practice and stand as a model where progressive clinical variation and difficulty can be presented [28]. Studies also show that VPs are a cost-effective way to teach and assess clinical skills and clinical reasoning among medical students [29].

Other studies indicate that participation in education using VPs may bridge the gap between experimental learning (learning by doing) and information representation (lectures) and consequently achieve a variety of learning outcomes [22,27]. Studies have also shown that VPs can enhance training of essential nursing skills and knowledge [30]. Although the research surrounding best practice of VPs is in its infancy [21], there are some studies that suggest that new learning technologies such as VPs and virtual reality may be useful to support nursing education. A challenge with most of the reported studies is their focus on the medical curricula [31,32]. There is a therefore an urgent need to investigate how to model VPs for nursing education.

Unfortunately, research on these learning technologies has not kept pace with the rapid technological development [27] and often lacks a theoretical foundation. The lack of empirical base in research lies at three levels: (1) the VPs reported in the literature have been created without an explicit and consistent underlying theoretical model, making it difficult for researchers and practitioners to build on the findings, (2) while a learning activity encompasses more than the VPs, we could not find that aspect systematically presented, and (3) there are different types of VPs and therefore a need to clearly understand the strengths and weaknesses of each type.

### Objective of the Study

The aim of this study was to (1) develop a theory-anchored model for developing virtual patients in nursing education, (2) investigate how VPs could be instantiated as a learning activity, and finally (3) explore the students’ perceived usefulness of virtual patients created from the developed model. To reach this aim, three research questions were asked:

1. What aspects of clinical reasoning should be present in a virtual patient nursing model?
2. How should the virtual patient nursing model be represented as a learning activity?
3. How do the nursing students perceive the usefulness of virtual patients as an artefact of the developed models?

## Methods

### Modeling of the Aspects in a Virtual Patient

The process of modeling started by a review of existing theoretical frameworks reported in the literature and in actual use when designing learning and assessment activities in nursing education. The 3^rd^generation nursing process model, the Outcome-Present State Test (OPT) model [11,33], was chosen because it is widely used internationally and was relevant for the focus of the study. This theoretical framework is outcome driven and builds on earlier versions of the nursing process. The OPT model provides structure for the iterative clinical reasoning necessary for contemporary nursing practice. The OPT model emphasizes reflection, outcome specification, and tests for judgment within the context of the individual patient story. It provides a way for nursing students to frame and attribute meaning to patient stories while concurrently considering relationships among diagnoses intervention and outcomes, with attention to evidence-based nursing and the decision-making process [34]. Essential processes contained within the OPT model are reflection, framing, cue logic, testing, decision making, and judgment [33]. An essential part of the OPT model is the emphasis on framing a situation based on the story of a client. The OPT model uses the facts associated with the client’s story to frame the context, content, and major issues for clinical reasoning. The patient stories are a key element of clinical reasoning [11,33].

The virtual patient nursing model was then, in an iterative manner, developed and optimized. Validation of the model was performed with faculty both in terms of relevance of chosen theories but also applicability of aspects represented in the model for the nursing education context.

### Representation of the Virtual Patient Model as a Learning Activity

Curriculum integration plays a key role in the implementation of new learning technologies [21]. In order to translate the virtual patient Nursing Design Model (vpNDM) to a learning activity, we chose the context of a second year undergraduate nursing course at a university in Sweden. The course content includes topics from nursing and medicine, and theory with practice. A large part of the course is based in a clinical setting.

Subject matter experts and educational researchers reviewed which intended learning outcomes in the course plan were suitable to be addressed using virtual patients. The faculty group then defined learning goals for the learning activity based on both the chosen intended learning outcomes and the developed vpNDM. The goal for the student while working with the VP cases was based on the nursing process, training nursing documentation, and nursing diagnosis. Students must increase knowledge of how to frame a patient story, identify and analyze it, and then document and evaluate it. In other words, they are trained to develop a nursing care plan based on the individual patient’s specific needs. An additional aim was to describe nursing interventions related to the nursing needs of the patient and to practice using measurement instruments for the assessment.

Based on the defined goal and requirements, a multidisciplinary team of subject matter experts, nurse educators, educational researchers, and clinical active nurses created two VP cases. Both cases were a loose amalgamation of actual cases experienced by the clinical team members.

### Authoring of the Virtual Patients

#### Method

We used the Virtual Interactive Case system (VIC) as a prototyping tool to create the VPs. VIC is a Web-based application using Adobe Flash. The VPs were accessible to the students through the university’s online course management system. The navigation was global linear, which means that there was a linear process but the user could freely navigate between the different sections of the VP [25]. The answers given by the VP were a combination of text and multimedia (images, sounds) [35].

**Figure 1:**
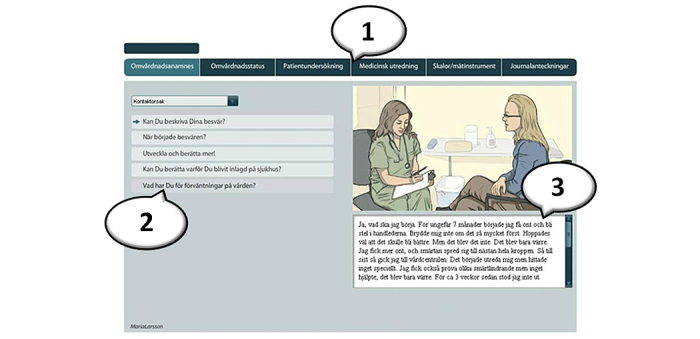
Screenshot of the system (VIC) used to author virtual patients.

A common model of interaction in VPs is the presence of long menus of questions, formulated by the author of the patient case. Students choose from a wide range of questions about different issues, and part of the task is to be able to select relevant questions. Figure 1 shows how students can navigate and decide on different options (1) containing the patient’s past history, the patient’s perception of the current situation, physical examination, medical investigation, scales and measuring instruments, and patient journal records. (2) Within each option, the student can then select among a number of questions or inquiries. (3) The answer is then presented as text, image, movie, or sound file.

In this study, we created two VPs. The first case was a woman in her 40s who was admitted to a ward with a 6-month history of pain related to joint inflammation (rheumatoid arthritis). She had several different nursing diagnoses related to chronic pain and readiness for enhanced comfort, impaired physical mobility, insomnia, and risk for impaired skin integrity. The second VP was an older man with type 2 diabetes mellitus. He was admitted to the ward with heart failure, hyperglycemia, and an acute diabetic foot. The nursing diagnosis in this VP case was related to excess fluid volume, impaired gas exchange, risk for unstable blood glucose level, ineffective self-health management, deficient knowledge, and impaired skin integrity.

#### Participants

The participants (N=102) were undergraduate nursing students whose age ranged between 20-53 years (mean age 23 years). The gender distribution was 12.5% male and 87.5% female. The VPs were integrated in the regular course as mandatory self-study learning.

The participants were free to take part in the study, and their anonymity and confidentiality were respected. The local ethical board approved the study.

#### Questionnaire

An online self-administered questionnaire developed by the eViP (electronic virtual patients) project team [36] was adjusted to fit nursing education. The questionnaire evaluated the students’ experiences of learning while working with virtual patients, focusing on the development of clinical reasoning skills. The questionnaire contains 17 items clustered into five subsets. The subset covered areas such as authenticity of patient encounter and the consultation, professional approach in the consultation, coaching during consultation, learning effect of consultation, and overall judgment of case workup. Responses were on a 5-point Likert scale (strongly disagree to strongly agree) as well as additional free text open-ended questions [36,37]. The participants answered the questionnaire after completing the two VPs. Completion of the survey was a voluntary part of the study.

#### Analysis

Descriptive statistics were performed and percentages reported. Cronbach alpha was calculated to provide an overall reliability coefficient for the set of questions included in the study. Item-total statistic was examined to check if any item was inconsistent with the others and could thus be discarded. Item-item correlation matrix was observed to examine the correlation between the items. “Strongly agree” and “agree” were combined to create a positive response.

## Results

### The Virtual Patient Nursing Design Model

#### Overview

**Figure 2:**
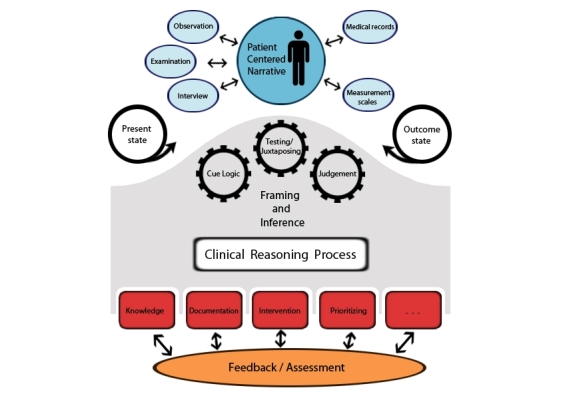
The three layers in the virtual patient nursing design model.

The model we produced (Figure 2) is based on and grounded in the 3^rd^generation nursing process, using the OPT model [11,33]. This model describes the elements that should be part of a VP meant for nursing education. The vpNDM model is composed of three main layers.

#### Layer 1: Patient-Centric Data Collection

The first layer includes different methods for collecting data on the patient. By capturing the patient story, the nurse obtains the necessary data to support the identification of nursing care needs. An essential part of nursing involves listening and understanding the meaning in a patient encounter. To capture the patient-centered narrative, the learner is given the opportunity to collect both subjective and objective data from the VP.

In order to capture the patient story, nurses seek information from various sources, for example by conversations or interviews with the patient or their relatives, physical examinations, other health care professionals’ evaluations, and patient records. By interviewing the patient, the nurse obtains a history of the patient’s past and present health. The past health history describes the patient’s previous health status and living conditions while the nursing status describes the current status and factors affecting the current nursing care.

In order to increase authenticity while students interact with the VP, the patient’s answers are provided in informal “spoken” language—breaks and emotional expressions are included as part of the answer. As in real life, the patient does not always directly answer the question asked. The question might be misunderstood or the patient chooses not to answer the question at all.

Physical examination provides important information for nurses to understand the patient’s situation. Physical examination is an assessment of the physical and mental status of the patient. In the box “Observation”, the student can gather data (assessment) by observation and inspection of the patient. It is also possible for learners to have access to clinical investigations conducted by other professionals, such as laboratory data and radiology reports. In these VP cases, the result was presented through text and images. The model does allow the use of other types of media such as video clips, but this option was not used in our study.

The box “Measurement scales” includes different assessment instruments used in nursing to systematically collect information to support judgments and decisions. Commonly used measuring scales are the Downton Fall Risk index and the Minimal Nutritional Assessment (MNA) scales.

Collecting data from the patient is not solely the nurse’s responsibility. Different health professionals contribute with information to the patient’s medical records. Different care providers then use information collected and reported by different health professionals. For example, both the nurse and the physician perform physical examination and patient history taking, but they use different formats and analyze the data differently because of each profession’s patient care focus. Therefore the medical records from different health professions include valuable information that can help the nurse to understand the patient’s story and determine the strengths of the patient or assess the responses the patient exhibits or could potentially exhibit, as a result of a health problem.

#### Layer 2: Iterative Clinical Reasoning

After obtaining the patient-centered narrative, the learner starts to frame or derive the meaning and significant issues and identify the patient’s nursing care needs by identifying the present state and the desired outcome state. The interplay between the description of the present state and how to attain the outcome state will drive the reasoning. One approach is juxtaposing where specific outcome state criteria are compared with present state data. A test is then created to evaluate the gap between the two states. Through a process of cue logic, juxtaposing, and judgment, the students choose the appropriate interventions that will bring present state and the desired outcome state closer to each other.

At this stage, the students’ strategies are important. In VPs, this process is supported by didactic questions that simulate reflection and make thinking skills visible.

#### Layer 3: Measurable Outcomes

The VP model allows assessment of how the student collects and analyzes data in order to identify and frame nursing care needs and the student’s ability to document their reasoning process. The patient’s care management can also be exposed in terms of the student’s ability to plan for accurate intervention and accurate prioritizing of nursing actions. The model also allows assessment of knowledge levels and the student’s ability to reflect on some specific aspects.

### The Virtual Patient Nursing Activity Model

**Figure 3:**
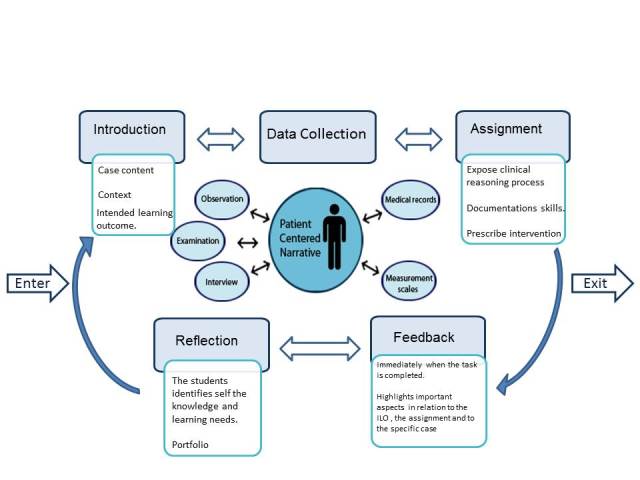
The different aspects in the virtual patient nursing activity model.

In order to be useful, the model needs to be contextualized in an educational setting with specific intended learning and/or assessment outcomes. The virtual patient Nursing Activity Model (vpNAM) contains both aspects that are necessary for a learning activity (see the light blue boxes in Figure 3) and is based on the intended learning outcomes specific instructional strategies. To illustrate the vpNAM, we applied it to the third semester of the undergraduate nursing education program at Karolinska Institutet. The goals of the learning activity in that semester were to define the patients’ nursing problems, create a nursing plan, train on documentation, and practice how to prescribe evidence-based nursing intervention.

When the vpNAM is applied to the VP, it means that the student is first introduced to the patient and the aims of the learning activity. The student is then expected to collect relevant data to frame an understanding of the patient’s needs and also capture the patient’s story. After completing the encounter with the VP, the student is given formative feedback that the case author formulated in advance. The student uses the feedback to reflect and to identify further knowledge and learning needs.

### Students’ Perceptions of the Virtual Patient Learning Activity

**Figure 4:**
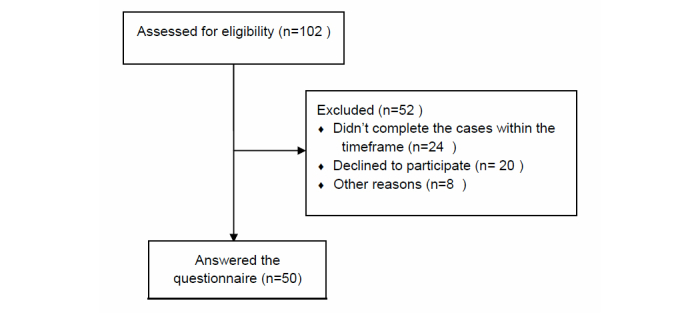
Flowchart showing the recruitment process of the participants.

We investigated how the VPs, based on the developed models, were perceived by nursing students. After completing two VPs, the participants answered an adapted version of the eViP questionnaire [36] adjusted to fit nursing education. All the students in the course (N=102) were invited to participate in the study. We then excluded those that either did not complete the cases within a given timeframe (first case in 3 weeks and second case in 2 weeks) or chose not to participate in the study. Figure 4 summarizes the process of recruitment of participants.

The questionnaire is a 14-item self-reported evaluation that explores five domains: authenticity, professionalism, learning, coaching, and overall judgment. The Cronbach alpha is .88 (greater than .7), which indicates a high level of internal consistency of scale with this specific sample. The construct has a good reliability. Table 1 shows the proportion of students that answered “agree” and “strongly agree” to the statements. Perceived usefulness is presented in a relative manner in order to highlight strengths and weaknesses of the type of VP created.

**Table 1:** Descriptive statistics and analysis of students’ perceived usefulness (full sample N=50).

Questions		Agree		
		n	%	Perceived usefulness^a^
**Authenticity of patient encounter and the consultation**				
	Q1.While working on this case, I felt I had to make the same decisions a nurse would make in real life.	22	43	**+**
	Q2. While working on this case, I felt I were the nursing student caring for this patient.	24	47	**+**
**Professional approach in the consultation**				
	Q3. While working through this case, I was actively engaged in gathering the information (eg, history questions, physical exams, lab tests) I needed, to characterize the patient’s nursing problem.	41	80	**+++**
	Q4. While working through this case, I was actively engaged in revising my initial image of the patient’s problem as new information became available.	32	63	**++**
	Q5. While working through this case, I was actively engaged in creating a short summary of the patient’s problem using nursing terms.	26	51	**+**
	Q6. While working through this case, I was actively engaged in thinking about which findings supported or refuted each nursing diagnosis.	31	61	**++**
**Coaching during consultation**				
	Q7. I felt that the case was at the appropriate level of difficulty for my level of training.	31	61	**++**
	Q8. The questions I was asked while working through this case were helpful in enhancing my diagnostic reasoning in this case	24	47	**+**
	Q9. The feedback I received was helpful in enhancing my diagnostic reasoning in this case.	27	53	**+**
	Q10. The feedback I received was helpful to improve my ability to identify nursing problems and nursing diagnoses	25	49	**+**
	Q11. The feedback I received helped me improve my ability to prescribe nursing intervention.	23	45	**+**
**Learning effect of consultation**				
	Q12. After completing this case, I feel better prepared to confirm a nursing diagnosis and exclude differential diagnoses in a real life patient with this complaint.	29	60	**++**
	Q13. After completing this case, I feel better prepared to care for a real life patient with this complaint.	23	45	**+**
**Overall judgment of the case workup**				
	Q14. Overall, working through this case was a worthwhile learning experience.	38	74	**+++**

^a^The level of perceived usefulness is indicated as medium (+), high (++), and very high (+++).

## Discussion

### Principal Findings

#### A Design Blueprint for Virtual Patients in Nursing Education

The aim of this study was (1) to develop a theory-anchored model for creating virtual patients in nursing education, (2) to investigate how VPs could be instantiated as a learning activity, and finally (3) to explore undergraduate nursing students’ perceived usefulness of two virtual patients.

We chose a theory-anchored design blueprint (Figure 2) that provides a structure for the authoring of virtual patients. The virtual patient Design Nursing Model (vpDNM) builds on the 3^rd^generation nursing process and is composed of three main layers. Layer 1 focuses on patient-centric data collection, Layer 2 is about iterative clinical reasoning, and Layer 3 covers measurable outcomes. The nursing process has provided structure and thinking in nursing education since the 1950s, and nurses recognize themselves in this way of thinking and working. However, the nursing process has evolved over time. While the first generation focused on problems and processes, the second generation emphasized diagnosis and reasoning. Finally, the third generation highlighted reflection, outcome specification, and testing given a patient’s story [11]. The developed design model reflects the nursing process (observation, assessment, nursing diagnosis, nursing prescription, planning, implementation, and evaluation) because it is recognized as a guideline for nurses in their clinical practice. However, the OPT model differs from the traditional nursing process by highlighting clinical reasoning. It is built on a foundation of reflective judgment. It honors the holistic nature of nursing and approaches patients’ situations in terms of outcomes. It identifies the thinking skills and thinking strategies involved in clinical decisions and judgment.

A further advantage is that this model can be used with several taxonomies used in nursing that can provide the content for clinical reasoning [11]. Choosing a different theoretical basis would probably have had an impact on the vpDNM by highlighting other aspects. We acknowledge that a model could hide the complexity of people’s health care needs that require the collective competence, skills, knowledge, and actions of different professions. Health care professionals share competencies, knowledge, and skills, but they also make unique contributions to the patient’s care [38]. As different professions contribute with different knowledge and skills to the health care environments, the different educational program has to focus on different learning goals. This is why this model is needed for nursing education, but also all contextualized models should be revisited when addressing other health care professions.

In the proposed design model, the nursing process has most influence on Layers 1 and 2. In Layer 1, it influences the way patient data are collected and what type of clinical data was made available. The purpose of data collection is to capture the patient-centered narrative. The learner is given the opportunity to collect both subjective and objective data from the VP. The third generation nursing process also had a substantial effect on Layer 2. In Layer 2, the emphasis is on reflection, outcome specification, and testing given a patient’s narrative.

The vpDNM is intended to work as guideline for teachers when they create VPs. The model will also allow the systematic development of different types of virtual patients from a common model and thereby create opportunities for sharing pedagogical designs across technical solutions. Finally, the model might help provide a basis for research that builds on shared empirical findings and best practices.

#### Virtual Patients in a Learning Activity

An activity model was built using the vpDNM combined with a nursing educational context, specifically, the third semester in an undergraduate nursing program. The intended learning outcomes informed the modeling process (Figure 3). The vpNAM we developed reinforces the increased understanding that VPs are note merely an object/tool but rather a learning activity. The proposed learning activity takes into account the core drivers of learning in medical simulation, which are scenario-based learning, task deliverables, trigger events, data collection guidance/instruction, feedback, and reflection [39]. The findings indicate that a learning activity using VPs is similar to other types of simulation-based learning opportunities that offer the potential to investigate constructive alignments between different types of simulation modalities for both learning and assessment. It also creates an incentive to further investigate the value of VP affordances in relation to the learning objectives and competencies.

Computer-based education and simulation have been proposed as an instructional method to develop nursing students’ ability to translate and apply theoretical knowledge in a clinical context [9]. The vpNAM contributes to systematizing the curricular integration that plays a key role in the implementation of new technologies and thereby ensures that the VPs are used effectively and consistently [21].

#### Students’ Perceptions of Global Linear Virtual Patients

We investigated the nursing students’ experiences of learning while working with virtual patients, created using the vpNDM and vpNAM, focusing on the development of clinical reasoning skills. The aspects covered authenticity of patient encounter and the consultation, professional approach in the consultation, coaching during consultation, learning effect of consultation, and overall judgment of case workup. The global linear VPs were perceived by students as useful in terms being actively engaged in gathering patient information in order to characterize the patients’ nursing problem. Others useful aspects, but to a lesser extent, were the ability to actively engage in revising the patients’ problems as new information became available and the fact that VPs triggered diagnostic reasoning.

### Conclusions

Virtual patients adapted to the nursing paradigm could support nursing students’ development of clinical reasoning skills. Building on theory-anchored models for designing and implementing virtual patients can aid constructive alignment with intended learning and assessment objectives. Furthermore, this approach could strengthen the research in this field. Finally, nursing students perceive the strengths of global linear VPs mainly in the ability to support clinical data gathering and as a worthwhile learning experience, and to a lesser extent, in VPs that actively drive the reasoning process.

## Abbreviations

CR: clinical reasoning
eViP: electronic virtual patients
OPT: Outcome-Present State Test
VIC: virtual interactive case
VP: virtual patient
vpNAM: virtual patient Nursing Activity Model
vpNDM: virtual patient Nursing Design Model
